# Two New Putative Plant Viruses from Wood Metagenomics Analysis of an Esca Diseased Vineyard

**DOI:** 10.3390/plants9070835

**Published:** 2020-07-03

**Authors:** Nadia Bertazzon, Walter Chitarra, Elisa Angelini, Luca Nerva

**Affiliations:** 1 Council for Agricultural Research and Economics—Research Centre for Viticulture and Enology CREA-VE, Via XXVIII Aprile 26, 31015 Conegliano (TV), Italy; nadia.bertazzon@crea.gov.it (N.B.); elisa.angelini@crea.gov.it (E.A.); 2 National Research Council of Italy—Institute for Sustainable Plant Protection CNR-IPSP, Strada delle Cacce 73, 10135 Torino, Italy

**Keywords:** grapevine, potyvirus, bunyavirus, coguvirus, esca disease

## Abstract

The concept of plant as a holobiont is now spreading among the scientific community and the importance to study plant-associated microorganisms is becoming more and more necessary. Along with bacteria and fungi, also viruses can play important roles during the holobiont-environment interactions. In grapevine, viruses are studied mainly as pathological agents, and many species (more than 80) are known to be able to replicate inside its tissues. In this study two new viral species associated with grape wood tissues are presented, one belongs to the *Potyviridae* family and one to the *Bunyavirales* order. Due to the ability of potyviruses to enhance heterologous virus replication, it will be important to assess the presence of such a virus in the grapevine population to understand its ecological role. Furthermore, the association of the cogu-like virus with esca symptomatic samples opens new questions and the necessity of a more detailed characterization of this virus.

## 1. Introduction

Grapevine is one of the most important cultivated crops in Europe and studies on its genetic variability were performed to understand varietal characteristics, unveiling only a small part of the complex biodiversity observed within these species [1]. A further layer of biodiversity is contemplated when considering grapevine as an ecological unit formed by plant together with the strictly associated microorganisms, defined as the holobiont. From the genetic point of view this ecological unit is represented by an extended genome made by every genomes of each biological unit, defined as the hologenome [2,3,4]. Viruses, which are part of the holobiont, have a significant impact on ecology and evolution of every ecological niche and are the most abundant type of replicative entity on the planet [5]. Crucial developments in molecular biology and genomics had enabled the scientific community to explore a larger picture of the microcosmos by obviating the need to culture microbes thanks to the next-generation sequencing (NGS) technologies and leading to the description of complex microbiomes [6,7].

*Vitis* spp. host one of the largest number of viruses among cultivated crop species, which is probably linked to a very long history of domestication and coexistence, an extensive exchange of germplasm on a global scale and the vegetative propagation [8]. The effects of some viral diseases on grapevine yield and quality are defined as severe, inducing extensive modulation in physiological performances. This modulation can also lead to a modification of the secondary metabolism pathways which can affect accumulation of pigments and aromatic compounds producing in turn a modification of the organoleptic characteristics in the final product [9]. In spite the general way to look at viruses as pathological agents, they can also have positive effects on the host, inducing the so called cross-talk, which can help the plant during stressful events [10] or to ameliorate organoleptic characteristics of wines [11].

Among grapevine trunk diseases (GTDs), esca syndrome is one of the most complex, characterized by simultaneous infection of several fungi, which lead to important reduction in yield and quality of fruits and wines [12]. The site in which the syndrome occurs is the main trunk, where the fungi invade the xylem vessels, produce toxic metabolites and cause many physiological changes which then lead to the induction of the tiger striped leaves, the hallmark of esca syndrome [13,14]. In a previous study we characterized the culturable mycobiome associated to esca symptomatic and asymptomatic plants and, to better characterize the fungal isolates we also described the associated mycovirome [7]. To understand the interactions occurring among a plant showing esca symptoms and the associated microbiota a metatranscriptomics approach was used, which allowed us to identify several viral genomes occurring in the woody tissue. Among these genomes two new putative plant viruses were identified, which could have an impact on plant physiology and could play a role in esca syndrome development.

## 2. Materials and Methods

### 2.1. Plant Material

The present study was conducted in an experimental vineyard of CREA - Research Centre for Viticulture and Enology (CREA-VE) located in Spresiano (TV), Veneto region, Italy (elevation is 56 m a.s.l and a warm temperature climate) [15]. Samples were collected from 20 years old grapevine plants cultivar Glera grafted onto SO4 rootstock on which the characterization of the culturable fungal endophytes was already performed [7].

Vineyard was visually monitored, plant by plant, to determine sanitary status and presence of esca-related symptoms for 4 years. Wood tissue was collected from 9 continuously asymptomatic plants (AS) and from 9 plants continuously symptomatic (SY) (tiger striped leaves and/or apoplexy) which showed symptoms at the collection date (16 September 2017). The wood tissue was sampled using a sterile corer, which allowed extracting a transversal wood fragment from the main trunk at the crown ramification level. Wood fragments were stored in sterile 50 mL conical tubes at −80 °C until further analysis.

### 2.2. RNA Extraction and Sequencing

Extraction of RNA was performed by making 3 pools of samples from 3 plants for each condition (AS and SY). To obtain total RNA from wood tissue a modified version of the protocol published by Gambino and co-authors in 2008 [16] was used. Briefly, wood samples were reduced to a very thin powder using mortar, pestle and liquid nitrogen, then 5 mL of pre-heated (65 °C) extraction buffer (2% CTAB, 2.5% PVP-40, 2M NaCl, 100 mM Tris-HCl pH 8.0, 25 mM EDTA pH 8.0 and 2% of β-mercaptoethanol added just before use) were added in 15 mL conical tubes, mixed with 150 mg of wood sample and incubated for 10 min at 65 °C. An equal volume of chloroform:isoamyl alcohol (24:1 v/v) was added, the tube was mixed vigorously by hand shaking and centrifuged at 8000× *g* for 20 min at 4 °C. Supernatant was recovered, transferred to a new 15 mL conical tube to perform a second chloroform:isoamyl alcohol extraction. The supernatant was retained, transferred in a new tube and amended with an equal volume of 100% isopropyl alcohol. Nucleic acids were allowed to precipitate at −20 °C for 30 min and then were collected by centrifuging at 8000× *g* for 40 min at 4 °C. The pellet was then washed with 70% ethanol, dried and resuspended in 250 µL TE buffer (10 mM Tris, 1 mM EDTA). Total RNA was recovered using the Spectrum plant Total RNA kit (Merck KGaA, Darmstadt, Germany): 500 µL of Binding buffer were added to the 250 µL of total nucleic acid and loaded onto the binding column. Then, washing steps were doubled (2 times Wash buffer 1 and 4 times Wash buffer 2) and final elution was performed in 40 µL. Quantity and quality of RNAs were checked using NanoDrop One apparatus (Thermo-Fisher Scientific, Waltham, MA, USA) and by running a 1% agarose gel in 0.5 % TBE buffer (45 mM Tris-borate, 1 mM EDTA).

The obtained samples were sent to Macrogen Inc. (Seoul, Korea) for rRNA depletion (Ribo-Zero™ Gold Kit, Epicentre, Madison, WI, USA), cDNA libraries construction (TrueSeq total RNA sample kit, Illumina) and sequencing by Illumina Novaseq technology with an output of 100M paired-end reds of 100 bp for each library.

### 2.3. Sequence Assembly and Analysis

*De novo* assembly from total-RNA sequencing was performed using high quality and cleaned reads selected using Trimmomatic [17]. For contigs assembly Trinity (version 2.3.2) was used [18], then blastx (version 2.6.0+) from the BLAST suite was used to search conserved viral proteins among the assembled contigs using a custom build reference database with viral sequences [19]. Alignments of reads against identified viral contigs were performed using BWA 0.7.15-r1140 [20] and SAMtools 1.3.1 [21]. Coding open reading frames (ORFs) were detected with ORF Finder (http://www.ncbi.nlm.nih.gov/gorf/orfig.cgi), blasted against the nr NCBI databases, and then the deduced protein molecular weight was calculated using the ExPASy online tool (https://web.expasy.org/compute_pi/). Conserved domain search was performed using the CDD/SPARCLE tool from NCBI [22].

To confirm that the viral contigs were not artifacts, quantitative RT-PCR (qRT-PCR) was performed for each RNA-dependent RNA polymerase (RdRP). First, cDNA was synthetized from total RNA following the manufacturer instructions provided for the High-Capacity cDNA Reverse Transcription kit (Thermo-Fisher Scientific, Waltham, MA, USA). Specific primers (Table S1) and iTaq universal SYBR Green supermix (Bio-Rad, Hercules, CA, USA) were then used in a CFX-96 (Bio-Rad) apparatus. Amplified fragments were sequenced to confirm the viral sequence. To further confirm the RNA nature of identified viruses and to avoid identification of endogenized viral sequences, the same qRT-PCR protocol was performed on all the infected isolates, but using as template RNase-treated DNA as previously reported [23].

The occurrence of identified viruses was assessed on grapevine samples collected in 2012–2019 and others collected in 2005 that were stored in the CREA-VE collection at −80 °C as total RNA extracts. RNA was extracted from the phloem tissue of woody canes or leaves according to Bertazzon et al. 2017 [24] and tested for the presence/absence of selected viruses as above described. An endogenous plant gene (ubiquitin) was co-amplified to check for archival RNA extract quality [11]. The core conserved part of each RdRP identified was used for multiple sequence alignments using MUSCLE [25] and then phylogenetic inference was performed using the maximum likelihood methodology in IQ-TREE [26]. Statistical analysis for each clade was carried out through bootstrap analysis with 1000 replicates.

### 2.4. Mechanical Transmission to Herbaceous Host

An attempt to transmit the two identified viruses to herbaceous host ***Nicotiana benthamiana*** and ***Chenopodium quinoa*** was performed. Homogenates of virus-infected plants were rubbed on the leaves surface of the two herbaceous hosts. After 7 and 15 days inoculated and non-inoculated leaves were harvested to extract RNA and test with qRT-PCR the presence of viruses.

## 3. Results

### 3.1. Sequencing Results and Viral Identification

The present virome analysis performed on the metagenomics samples of 6 libraries from grapevine wood tissue (3 esca asymptomatic and 3 esca symptomatic) retrieved over 150 contigs carrying RdRP signature motifs [27]. Here, we will focus our analyses only on those putative viruses which probably infect the plant, some of which fall into new taxonomic groups and which display as first hit in the nr protein databases a confirmed plant virus. The first virus identified was a contig of 11,032 nt which shows similarity with several viruses belonging to the Potyviridae family and which was present in all the libraries as reported in Table S2. It shows a unique open reading frame (ORF) of 10,707 nt encoding for a polyprotein of 407.6 kDa. A search with CDD/SPARCLE found an RT-like domain (typical in RdRP of positive single-stranded RNA viruses), a HrpA-like RNA helicase (involved in RNA replication and ribosomal interaction), a potyvirus coat protein domain (hallmark of potyviruses), a potyvirus polyprotein domain, a peptidase C4 and a peptidase C6 as reported in Figure S1.

The second virus, detectable only in the three symptomatic esca samples as reported in Table S2, which showed high similarity to plant viruses, is composed by three gene segments which show similarities to viruses belonging to the Bunyavirales order. The first genome segment is 7396 nucleotides long and encodes for a protein of 256.2 kDa with a conserved RdRP domain (CDD/SPARCLE search identified a Bunya_RdRp super family domain) as reported in Supplementary Figure S1. The second genome segment is 2250 nucleotides long and encodes for a protein of 77.2 kDa. No conserved domain was found by the CDD/SPARCLE search, but blastp alignment highlighted a very high similarity with movement proteins of viruses belonging to the Bunyavirales. The last genome segment is 1665 nucleotides long and encodes for two proteins, one in the positive and one in the negative strand. The protein encoded by the negative strand shows a predicted molecular weight of 29.7 kDa and a conserved domain of Tenuivirus/Phlebovirus nucleocapsid protein (CDD/SPARCLE search) as reported in Figure S1. The protein in the positive sense has a predicted molecular weight of 20.6 kDa, no conserved domain found with CDD/SPARCLE search and no similarity with any other protein in the nr database.

### 3.2. Phylogenetic Placement of Identified Viruses

**Figure 1:**
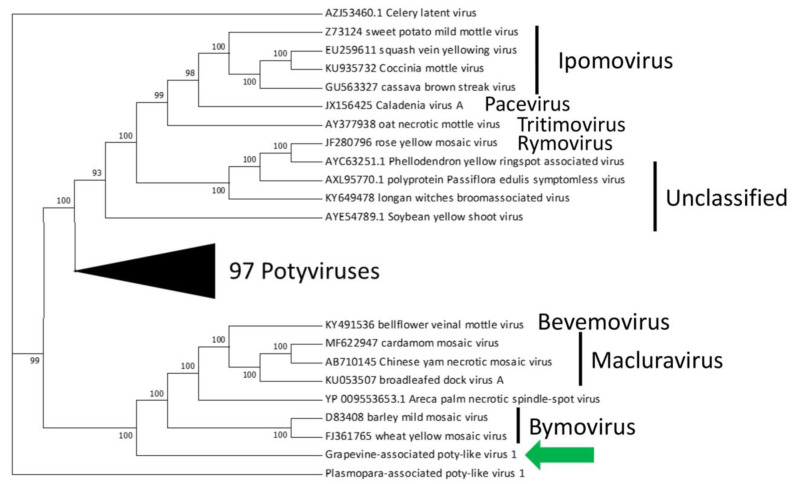
Phylogenetic analysis of a selected number of Potyviridae-like RNA-dependent RNA polymerases (RdRP). The newly identified virus is highlighted by the green arrow. Phylogeny was constructed by maximum likelihood algorithm and 1000 bootstrap replicates.

Blast search for the poty-like virus displayed that the first two hits in the nr database are macluraviruses (Narcisus latent virus—QDC21212.1 and Yam chlorotic necrosis virus—AWH61232.1) whereas the third match is a Bymovirus (Barley mild mosaic virus—BAA01742.1). For this reason sequences of potyviruses were used to build a phylogenetic tree. As reported in Figure 1, the virus identified in the present study falls in the clade where sequences of bymoviruses, macluraviruses and bevemoviruses are reported. Due to this similarity with potyviruses we proposed the name of Grapevine-associated poty-like virus 1 (GaPlV1) and its sequence is available in NCBI with the accession number MT353901.

**Figure 2:**
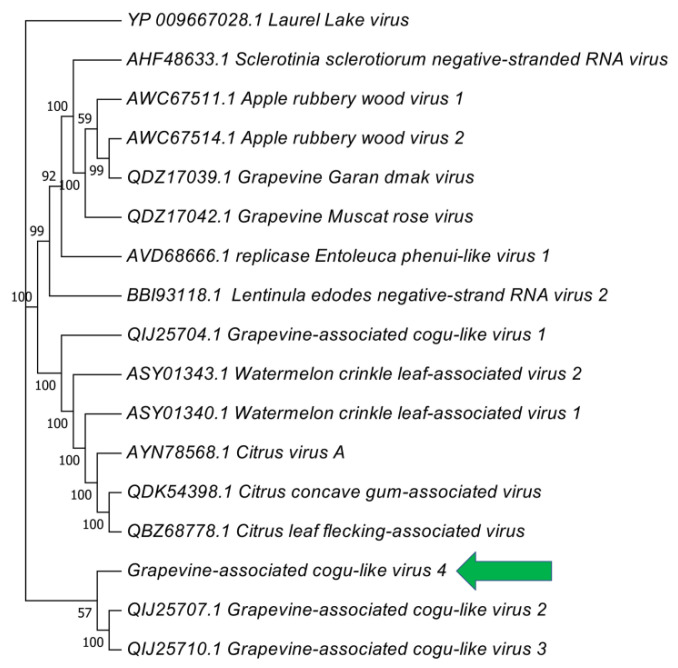
Phylogenetic analysis of a selected number of Coguvirus-like RNA-dependent RNA polymerases (RdRP). The newly identified virus is highlighted by the green arrow. Phylogeny was constructed by maximum likelihood algorithm and 1000 bootstrap replicates.

Searching for similarities in the nr database, the RdRP segment of the bunya-like virus displayed similarities with two recently reported cogu-like viruses (Grapevine associated cogu-like virus 2—QIJ25707.1 and Grapevine associated cogu-like virus 3—QIJ25710.1), with a Laulavirus (Laurel Lake virus—YP_009667028.1 in the Phenuiviridae family) and with a Coguvirus (Citrus concave gum-associated virus—QDK54399). As reported in Figure 2, the viral sequence identified groups with recently identified cogu-like viruses from mixed samples of grapevine leaves and Plasmopara viticola. Due to the close relativeness to the Coguvirus genus we proposed the name of Grapevine-associated cogu-like virus 4 (GaClV4). The sequences of the three genome segments were deposited in NCBI database with the accession numbers MT353902, MT353903 and MT353904.

### 3.3. Epidemiological Analysis and Mechanical Inoculation

The occurrence of the two newly identified viruses was investigated on 91 grapevine samples belonging to 64 cultivars collected between 2012 and 2019 from commercial vineyards and germplasm collections of the major grapevine growing regions around Europe (Table 1). Fifty-seven samples shown to harbor GaPlV1, while none of the analyzed samples was found infected with GaClV4. The poty-like virus was detected in samples collected from all the countries considered, although with different incidence. Excluding Portugal and Bulgaria, which showed low presence of the virus, an impressive mean infection rate (63%) was registered in the other countries. Further surveys performed on 14 samples collected in 2005 from different European countries revealed the presence of GaPlV1 on 6 samples, indicating that the coexistence of this virus with grapevine has lasted for more than 15 years.

The mechanical inoculation on herbaceous plants revealed that none of the two viruses were able to replicate in such hosts at both 7 dpi and 15 dpi.

## 4. Discussion

Viruses are the most frequent causal agents of emerging infectious diseases in crops [28,29], and are responsible of yield and quality losses that lead to important economic and social impacts [30]. As previously mentioned, to date, a vast number of viruses were reported as able to replicate in grapevine, and many are responsible for physiological changing which lead to decreased production or modification of the organoleptic composition in the final products [9]. Outcome of new viral entities are often detected in vineyards with several unpredictable features, such as limited understanding of the interaction with their host, their etiology, poor knowledge of route(s) of infection and dispersal, and lack of biological and molecular tools to study the virus ecology. In spite of all these obstacles, it is important to deepen the knowledges of new reported viruses, since, as already demonstrated in many cases, they can play important roles in defining the interaction among the plant, the associated microbes and the environment [10,11,24,31]. In specific, here we have identified two new viruses and now we are going to discuss their potential impact on plant physiology that can go further than a simple pathogen-host relationship.

Potyviruses are a large and agriculturally important group of plant viruses which can lead to severe losses in crop quality and yield, as in the case of Potato virus Y (PVY), Plum pox virus (PPV), Soybean mosaic virus (SMV) or Zucchini yellow mosaic virus (ZYMV) [32]. Interestingly, the emergence of potyviruses is dated more than 6600 years ago and it is commonly accepted that human agricultural activities has made an important contribution to the dispersion and evolution of this group of viruses [33]. One of the most interesting potyviral proteins is the helper component-proteinase (HC-Pro) which is able to interact with both viral and host plant proteins [34]. Among plant proteins the interaction between HC-Pro and the HUA enhancer 1 (HEN1) is at the basis of virus ability to suppress the plant endogenous silencing machinery by decreasing the accumulation of small interfering RNA (siRNA) [35]. As a consequence, the infection by a potyvirus can have a broad range of effects, inhibiting the normal development and functioning of the endogenous micro RNA (miRNA) and by trans-activating the replication of heterologous viruses [36,37]. To date no potyviruses were reported in grapevine, with the exception of Plasmopara associated poty-like virus (PaPlV1) [38] which, as reported by the authors, is most probably a fungal virus, and hence the virus here described represents the first virus belonging to this group able to infect grapevines. It is worth nothing that, a potyviral-like coat protein has integrated into the grapevine genome suggesting that infection by an unknown ***Potyvirus*** probably occurred in the past [39]. The relevance of this discovery is related to, the wide diffusion and the possible effects on heterologous viral replication that could explain, at least in part, the broad susceptibility of vines to viral infection. It is worth noting that, as reported in Table 1 GaPlV1 shows a high infection rate among countries of the Mediterranean: Italy, Spain, Greece and Croatia show an average diffusion above the 70%. In addition, differently from what recently reported for the poty-like virus isolated in the mixed sample of grapevine and *P. viticola* [38], the virus identified in the present work shows a more strict relationship with plant viruses. In specific, when comparing the sequence of our virus with the previously reported PaPlV1 we were able to find more conserved domains and moreover, looking at the phylogenetic analysis we can observe that GaPlV1 fall into the clade of *Macluravirus*, *Bymovirus* and *Bevemovirus* while PaPlV1 is far from any known potyviral family.

Coguviruses are a group of recently characterized plant viruses, closely related to phenuiviruses and inside the *Bunyavirales* order [40]. Interestingly, as also reported for other three cogu-like viruses [38], the virus identified in the present work seems to have a three-segmented genome. This is contrary to what happens for proper coguviruses, where the Nc and the putative MP are expressed from the same RNA segment in ambisense orientation, but it is consistent with other phenui-like viruses which are reported as three-segmented genome [41,42]. Another fascinating observation comes from the fact that in the *Coguvirus* genus there is the Citrus concave gum-associated virus, which was recently demonstrated to play a role during the development of a wood disease named as citrus disease concave gum-blind pocket (CG) [43,44]. In addition, other two viruses in the same clades were associated with wood diseases in apple [45,46]. Recently two new viruses belonging to the family Phenuiviridae were reported in grapevine and fully described [47]. It is interesting to note that, in our samples only the esca symptomatic vines displayed the presence of GaClV4. Looking at the epidemiology, despite the absence of information about the esca symptomatology on analyzed samples, we were surprised to get all the 91 cDNAs screened negative for GaClV4 infection. There are two possible explanations for the observed result: i) the virus is not infecting the plant but a fungal endophyte hosted in the woody tissue, or ii) the ability of GaClV4 to reach the phloem tissue is limited and hence we were unable to find it in the cDNA collection. The latter point is explained by the common method for viral detection in grapevine plants: the tissue from which usually RNAs for viral detection are extracted is the phloem of lignified branches. Therefore, if the virus is unable to reach such tissues we are unable to check its presence in the analyzed samples. Interestingly, fungal viruses able to enhance their virulence against the plant host were already reported [48,49,50]. Finally we also evaluated the possibility to infect herbaceous host using homogenates from leaves of infected plants, but we were unable to transmit none of the viruses. This result is not surprising since the vast majority of the known grapevine viruses are not transmissible to herbaceous plants [51,52,53,54,55,56]. Further studies are ongoing to understand if the presence of GaClV4 can have a role in esca syndrome development by both infecting the plant or any fungal endophyte hosted by the plant.

## 5. Conclusions

In the present work we reported for the first time two new viral species, one belonging to the *Potyviridae* family and one to the *Bunyavirales* order that are associated with grapevine plants. The biological impact of such viruses on the plant physiology is still to be elucidated but their relatedness to plant pathogenic viruses can help explaining the high susceptibility of *Vitis* spp. to viral infection (at least for the GaPlV1). To deepen this aspect some experiments on virus host-range are ongoing in controlled conditions. In parallel, further analyses are ongoing to understand if the cogu-like GaClV4 can play part in the development of esca syndrome, which leads to an important impairment of grape physiological performances and to a complex wood disease. To achieve this results wood metatranscriptome analysis are now under evaluation to observe plant, fungal and viral interactions.

## Figures and Tables

**Table 1:** Detection of GaPlV1 by qRT-PCR in different grapevine cultivars collected in 2012–2019 from several European countries. The number of GaPlV1-infected plants out of the total number of plants is reported for each country.

Country	Cultivar	GaPlV1-Infected/Total Samples
Italy	Bianco d’Alessano, Cesanese d’Affile, Chardonnay, Cornalin, Croatina, Glera, Gratena, Lambrusco Maestri, Lambrusco Salamino, Merlot, Nerello Mascalese, Pinot gris, Pinot noir, Primitivo, Traminer, Trebbiano toscano, Verdicchio, Vermentino	21/28
Portugal	Albariño, Cannonau, Chardonnay, Fernão Pires, Godello, Malvasia fine, Tinta barroca, Touriga Franca	1/10
Spain	Airen, Albariño, Fernão Pires, Macabeo, Parellada, Pedro Ximenez, Tinta barroca, Touriga Franca, Touriga National, Trepat	8/10
France	Chardonnay, Pinot gris, Pinot noir	5/10
Greece	Agiogirtiko, Assyrtiko, Kidonitsa, Kocifali, Roditis, Vilana	7/10
Bulgaria	Chardonnay, Dymiat, Brestovitsa, Pamid	1/4
Romania	Busuioaca de Bohotin, Galbena de Odobesti, Merlot, Rkatsiteli	4/5
Hungary	Franconia, Furmint, Harslevelu	2/5
Ukraine	Kokur Belji, Krasnostop zolotovsicij, Plecistik, Telebi koruk, Zimljansku cernj	4/5
Croatia	Malvasia, Plavac mali, Teran	4/4
**Total**	**64**	**57/91**

## Supplementary Materials

The following are available online at https://www.mdpi.com/2223-7747/9/7/835/s1, Figure S1: Genome representations for both Grapevine associated poty-like virus 1 (GaPlV1) and Grapevine associated cogu-like virus 4 (GaClV4). The blue lines represent open reading frames (ORFs), light blue lines are the CDD/SPARCLE recognized domains and arrows represent the reading sense, Table S1: List of primers (and a probe) used for detection of the new viruses we identified, Table S2: Number of reads and average depth for each virus and genome segment detected in the 6 analyzed samples.
