# Supplementary material for: Two New Putative Plant Viruses from Wood Metagenomics Analysis of an Esca Diseased Vineyard
Source: Plants (Basel). 2020 Jul 3;9(7):835. doi: 10.3390/plants9070835 (PMC7412230; doi:10.3390/plants9070835)
Supplement: Supplementary file 1 [file plants-09-00835-s001.pdf]

plants

MDPI

Communication

Two new putative plant viruses from wood metagenomics analysis of esca diseased grapevines

Nadia Bertazzon[1], Walter Chitarra[1,2*], Elisa Angelini[1] and Luca Nerva [1,2*]

[1] Council for Agricultural Research and Economics – Research Centre for Viticulture and Enology CREA-VE, Via XXVIII Aprile 26, 31015 Conegliano (TV), Italy
[2] National Research Council of Italy - Institute for Sustainable Plant Protection CNR-IPSP, Strada delle Cacce 73, 10135 Torino, Italy
* Correspondence: walter.chitarra@crea.gov.it; (W.C.)
luca.nerva@crea.gov.it; (L.N.)

Received: date; Accepted: date; Published: date

Abstract: The concept of plant as a holobiont is now spreading among the scientific community and the importance to study plant-associated microorganisms is becoming more and more necessary. Along with bacteria and fungi, also viruses can play important roles during the holobiont-environment interactions. In grapevine, viruses are studied mainly as pathological agents, and a huge number of species (more than 80) are known to be able to replicate inside its tissues. In this study two new viral species associated with grape wood tissues are presented, one of which belongs to the *Potyviridae* family and one to the *Bunyavirales* order. Due to the ability of potyviruses to enhance heterologous virus replication, it will be important to assess the presence of such a virus in the grapevine population to understand its ecological role. Furthermore, the association of the cogu-like virus with esca symptomatic samples suggests a possible involvement in syndrome development as also demonstrated for other viruses belonging to the same clade.

Keywords: grapevine, potyvirus, bunyavirus, coguvirus, esca disease

---

Supplementary Information

Table S1. List of primers (and a probe) used for detection of the new viruses we identified.

Primer name	Used for	Primer sequence	Target virus
VvPV1_1_For	qRT-PCR	GCCGCCACACTTGTCTACGT	GaPlV1
VvPV1_1_Rev	qRT-PCR	TGAAGCGAGGTTGGTCAGGA	GaPlV1
VvPV1_1_Probe	qRT-PCR	GCTGACACTATCTTTCCACGC	GaPlV1
VvPV1_264bpFor	RT-PCR	CACCTGTGACGAGATGGGCG	GaPlV1
VvPV1_264bpRev	RT-PCR	TCCACATTTGTATCCCCAATGCA	GaPlV1
VvBV1_For	qRT-PCR	AGCCAACCACACCACCACTG	GaClV4
VvBV1_Rev	qRT-PCR	GATTGTTCGGGGCGGCTGAA,	GaClV4
VvBV1_340bpRev (to be used with VvBV1)	RT-PCR	GGTGCGTCTGGTTCTGCTCT	GaClV4

plants

MDPI

Table S2. Number of reads and average depth for each virus and genome segment detected in the 6 analyzed samples.

	As1		As2		As3		Sy1		Sy2		Sy3	
	Reads number	Av. Depth	Reads number	Av. Depth	Reads number	Av. Depth	Reads number	Av. Depth	Reads number	Av. Depth	Reads number	Av. Depth
GaClV4 - RNA1	n.d.		n.d.		n.d.		6692	88	3253	44	4638	63
GaClV4 - RNA2	n.d.		n.d.		n.d.		2210	95	1069	48	2232	99
GaClV4 - RNA3	n.d.		n.d.		n.d.		809	49	414	25	967	58
GaPlV1	4566	41	5280	48	3619	33	20895	191	6617	61	11942	108

n.d.: not detected

Figure S1. Genome representations for both Grapevine associated poty-like virus 1 (GaPlV1) and Grapevine associated cogu-like virus 4 (GaClV4). The blue lines represent open reading frames (ORFs), light blue lines are the CDD/SPARCLE recognized domains and arrows represent the reading sense.

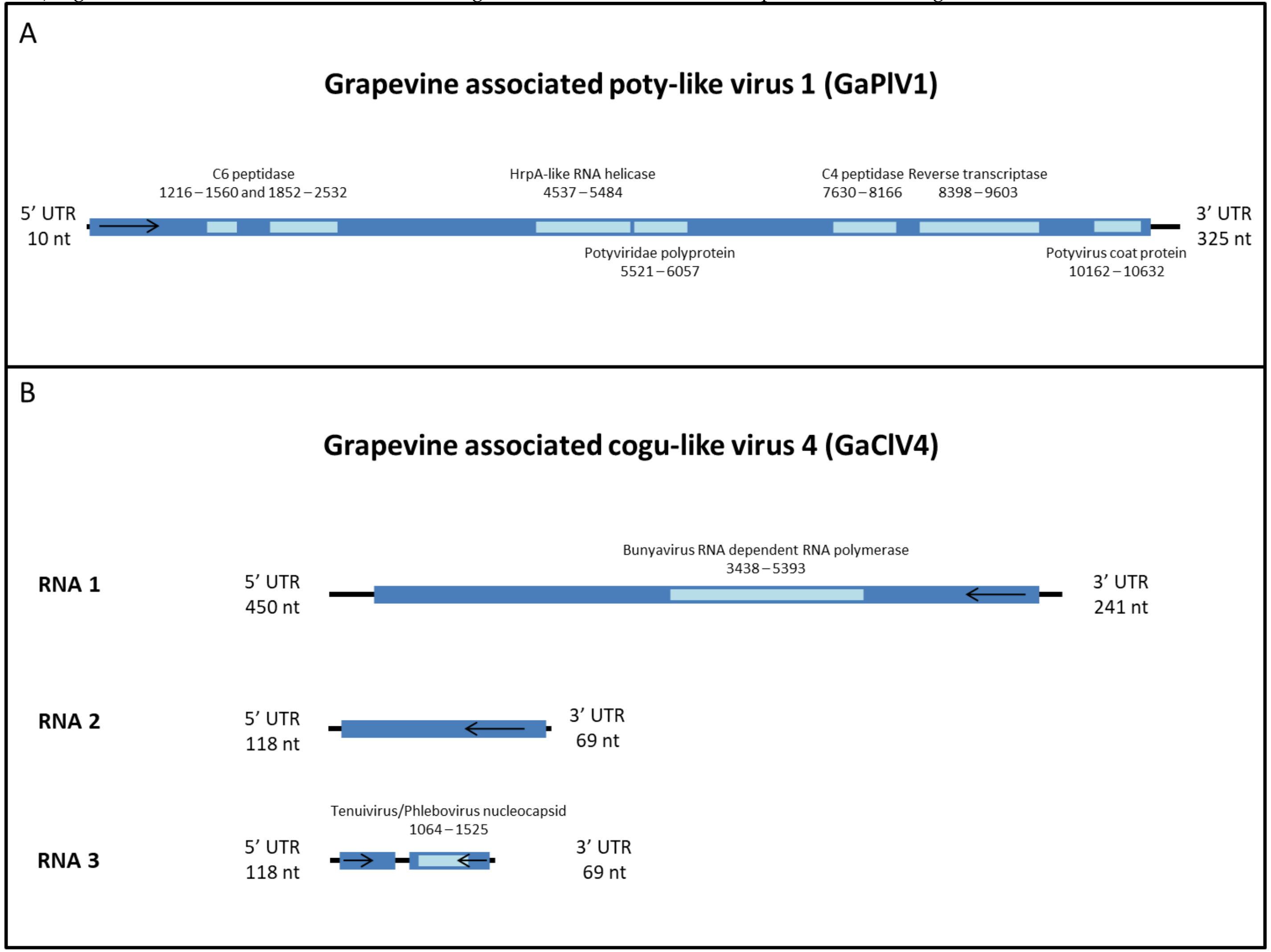

© 2020 by the authors. Submitted for possible open access publication under the terms and conditions of the Creative Commons Attribution (CC BY) license (http://creativecommons.org/licenses/by/4.0/).
